# A PCR assay to quantify patterns of HBV transcription

**DOI:** 10.1099/jgv.0.001373

**Published:** 2019-12-17

**Authors:** Valentina D’Arienzo, Andrea Magri, James M. Harris, Peter A. C. Wing, Chunkyu Ko, Claudia Orbegozo Rubio, Peter A. Revill, Ulrike Protzer, Peter Balfe, Jane A. McKeating

**Affiliations:** 1 Nuffield Department of Medicine, University of Oxford, Old Road Campus, Roosevelt Drive, Oxford, UK; 2 Institute of Virology, Technische Universität, München/Helmholtz Zentrum München, Germany; 3 Victorian Infectious Diseases Reference Laboratory, Royal Melbourne Hospital at the Peter Doherty Institute of Infection and Immunity, Melbourne, Australia; 4 Department of Microbiology and Immunology, University of Melbourne, Melbourne, Australia; 5 German Center for Infection Research (DZIF), Munich partner site, Munich, Germany; 6 Institute of Immunology and Immunotherapy, University of Birmingham, UK

**Keywords:** Hepatitis B virus, RNA, transcription

## Abstract

Hepatitis B virus (HBV) is the prototype member of the family *Hepadnaviridae* and replicates via episomal copies of a covalently closed circular DNA (cccDNA) genome of approximately 3.2 kb. The chromatinization of this small viral genome, with overlapping open reading frames and regulatory elements, suggests an important role for epigenetic pathways to regulate HBV transcription. However, the host pathways that regulate HBV transcription and the temporal nature of promoter usage in infected cells are not well understood, in part due to the compact genome structure and overlapping open reading frames. To address this we developed a simple and cost-effective PCR assay to quantify the major viral RNAs and validated this technique using current state-of-art *de novo* HBV infection model systems. Our PCR method is three orders of magnitude more sensitive than Northern blot and requires relatively small amounts of starting material, making this an attractive tool for assessing HBV transcription.

## Introduction

Hepatitis B virus (HBV) is a global health problem, with more than 250 million people chronically infected and at least 880 000 deaths per year from HBV-related liver diseases such as cirrhosis and hepatocellular carcinoma (WHO, Global hepatitis Report 2017, https://www.who.int/hepatitis/publications/global-hepatitis-report2017/en/). Currently available treatments suppress viral replication but are not curative, largely due to the persistence of the cccDNA transcriptional template in hepatocytes [1]. The recent success of antiviral drugs for treating hepatitis C virus infection has shown that curative therapies for hepatitis viruses are possible, providing a growing impetus to identify improved therapies for HBV [2].

HBV replication is primarily determined by the size of the cccDNA pool and its transcriptional activity [3]. cccDNA associates with chromatin and is frequently referred to as a viral mini-chromosome, where gene transcription is regulated by DNA methylation and epigenetic modifications to bound histones [4–6]. The chromatinization of this small episomal viral genome, with overlapping open reading frames and regulatory elements, suggests an important role for epigenetic pathways in regulating HBV transcription [7–9]. Recent studies highlight the role of host pathways in regulating the DNA epigenome of many viruses and indicate new pathways for anti-viral intervention [10, 11].

The HBV genome encodes two enhancers that activate four promoters to transcribe five major viral RNAs: 3.5 kb pre-core (pC), which encodes the HBV e antigen (HBeAg); 3.5 kb pre-genomic (pg) RNA encoding the capsid protein and polymerase; 2.4 kb preS1 and 2.1 kb preS2 RNAs, which encode the surface glycoproteins; and 0.7 kb X RNA, which encodes the multi-functional hepatitis B virus x protein (HBx) [12]. These viral RNAs share the same polyadenylation site and thus have the same 3′-end sequence. Recent studies have identified several spliced RNAs whose functions are not well understood [13]. Epigenetic transcriptional control is largely defined by two mechanisms: methylation of gene promoters and modification of histones by methylation or acetylation within enhancers, promoters and gene bodies.

However, our understanding of HBV promoter usage and the patterns of viral transcripts in different stages of chronic hepatitis B disease is limited [14], with the majority of studies only quantifying pgRNA as a marker of cccDNA transcriptional activity [15, 16]. The classical method to identify HBV transcripts is by physical visualization of RNA molecule size and abundance by Northern blot (NB) analysis. However, this approach is at best semi-quantitative and requires relatively large amounts of RNA, limiting its utility for studying viral transcripts in clinical samples. We have developed and validated a simple PCR-based quantification method to estimate the relative abundance of the major HBV RNAs.

## Methods

### Cell lines and HBV molecular clones

HepG2.2.15 cells and HepG2-NTCP cells [17] were maintained in Dulbecco’s modified Eagle’s medium (DMEM) supplemented with 10% foetal bovine serum (FBS), 2 mM L-glutamine, 1 mM sodium pyruvate, 50 U ml^−1^ penicillin/ streptomycin, and non-essential amino acids (all reagents from Thermo Fisher Scientific, Waltham, MA, USA). HepaRG cells expressing HBx under the control of a tetracycline-inducible promoter (HepaRG-TR-HBx) were provided by David Durantel (INSERM, Lyon) and cultured in Williams E medium supplemented with 10% FBS, 50 U penicillin/ streptomycin ml^−1^, 5 μg human insulin ml^−1^ and 5×10^−7^ M hydrocortisone hemisuccinate (Sigma). HepaRG-TR-HBx cells were treated with 1μg ml^−1^ of doxycycline to induce HBx expression and refed with fresh media every 3 days. All cells were maintained in a 5% CO_2_ atmosphere at 37 °C. Plasmids encoding HBV1.3 genotypes A2, B2, C2, D3 [18], H [19] and E [20] have been reported previously. Each plasmid was quantified by nanodrop and the inferred copy number μl^−1^ was calculated using an online tool (http://scienceprimer.com/copy-number-calculator-for-realtime-pcr).

### HBV genesis and infection

HBV ayw stocks were purified from a HepAD38 producer line as previously reported [17]. Wild-type (WT) and HBx-deficient (X^neg^) virus were reported previously [21] and purified from the extracellular media of HepG2 cells transfected with pHBV1.3 genomes. Virus was purified using centrifugal filter devices (Centricon Plus-70 and Biomax 100.000, Millipore Corp., Bedford, MA, USA) and stocks with a titre between 3×10^9^ and 3×10^10^ viral genome equivalents (vge) ml^−1^ were stored at –80 °C. HepG2-NTCP cells were treated with 2.5% dimethyl sulphoxide (DMSO) for 3 days and inoculated with HBV at an m.o.i. of 200 in the presence of 4% polyethylene glycol 8000. After 18-20 h the inocula were removed by washing with phosphate-buffered saline (PBS) and the cells were cultured in the presence of 2.5% DMSO. Secreted HBe and HBs antigen were quantified by EIA (Autobio, PR China).

### RNA isolation for cDNA synthesis

Total cellular RNA was extracted using an RNeasy mini kit (Qiagen) following the manufacturer’s instructions. To remove any residual HBV DNA, samples were treated with RNase-free DNasel (14 Kunitz units/rxn; Qiagen) for 30 min at room temperature. RNA concentration (μg ml^−1^) and quality (RIN score [22]) were determined (NanoDrop 1000 spectrophotometer, Thermo Scientific and 2100 Bioanalyzer, Agilent, respectively). cDNA syntheses were performed with 0.25–1 μg of RNA in a 20 μl total reaction volume using a random hexamer/oligo dT strand synthesis kit in accordance with the manufacturer’s instructions (10 min at 25 °C; 15 min at 42 °C; 15 min at 48 °C; Sensi-Fast, Bioline). Three additional cDNA kits were assessed following the manufacturer’s protocols: a PCR biosystem kit that uses a mixture of random hexamers and oligo-dT and the Ultra 2.0 (PCR Biosystems) and Superscript III (Life Technologies) kits that both use exclusively random hexamers. Completed reactions were inactivated at 85 °C for 5 min and stored at –70 °C.

### Quantitative PCR

All PCR reactions were performed using a SYBR green realtime PCR protocol (qPCRBIO SyGreen, PCR Biosystems) in a Lightcycler 96 instrument (Roche). The amplification conditions were: 95 °C for 2 min (enzyme activation), followed by 45 cycles of amplification (95 °C for 5 s; 60 °C for 30 s). A melting curve analysis was performed on the completed reactions to assess the specificity and purity of the amplicons (95 °C for 10 s; 60 °C for 60 s; followed by gradual heating from 60 to 97 °C at 1 °C s^−1^). DNase-treated RNA samples that had not been reverse-transcribed were amplified to verify the absence of residual DNA contamination. The sensitivity of PCR amplification of the pHBV1.0 ayw DNA was assessed using Applied Biosystems 7500 and Aligent MX3005P PCR machines, which provided comparable results. In addition, SYBR green kits purchased from Bioline or Life Technologies (Thermo Fisher) resulted in comparable amplification. Each of the PCR products was cloned into pCR2.1TA (Invitrogen) and used to test the specificity of the primers for their respective targets.

### Northern blotting

Samples were analysed as described previously [17]. Briefly, RNA from HepG2.2.15 cells, cultured with or without 2.5% DMSO, was extracted using Trizol Reagent (Life Technologies) and 10 μg of purified RNA was electrophoresed in a 1% MOPS agarose gel containing 2.2M formaldehyde. 18S and 28S ribosomal RNA species were visualized under UV light after electrophoresis to verify the amount of RNA loaded and to assess degradation. After denaturation (50 mM NaOH for 5 min). RNAs were transferred to a nylon membrane by capillary transfer using 20× SSC buffer. Membranes were washed and RNAs fixed by UV crosslinking. To detect HBV RNA, membranes were hybridized at 65 °C overnight with a digoxigenin-labelled DNA probe covering the entire HBV genome and visualized using a luminescent DIG detection kit (Roche).

### Statistical analyses

All analyses were performed using Prism 8 (GraphPad, La Jolla, CA, USA). Data are shown as means±SD; probabilities are indicated by *=*P*<0.05, **=*P*<0.01, ***=*P*<0.001 or ****=*P*<0.0001; and Bonferroni corrections are applied for multiple testing when appropriate.

## Results and Discussion

### Validating the PCR protocol

**Fig. 1:**
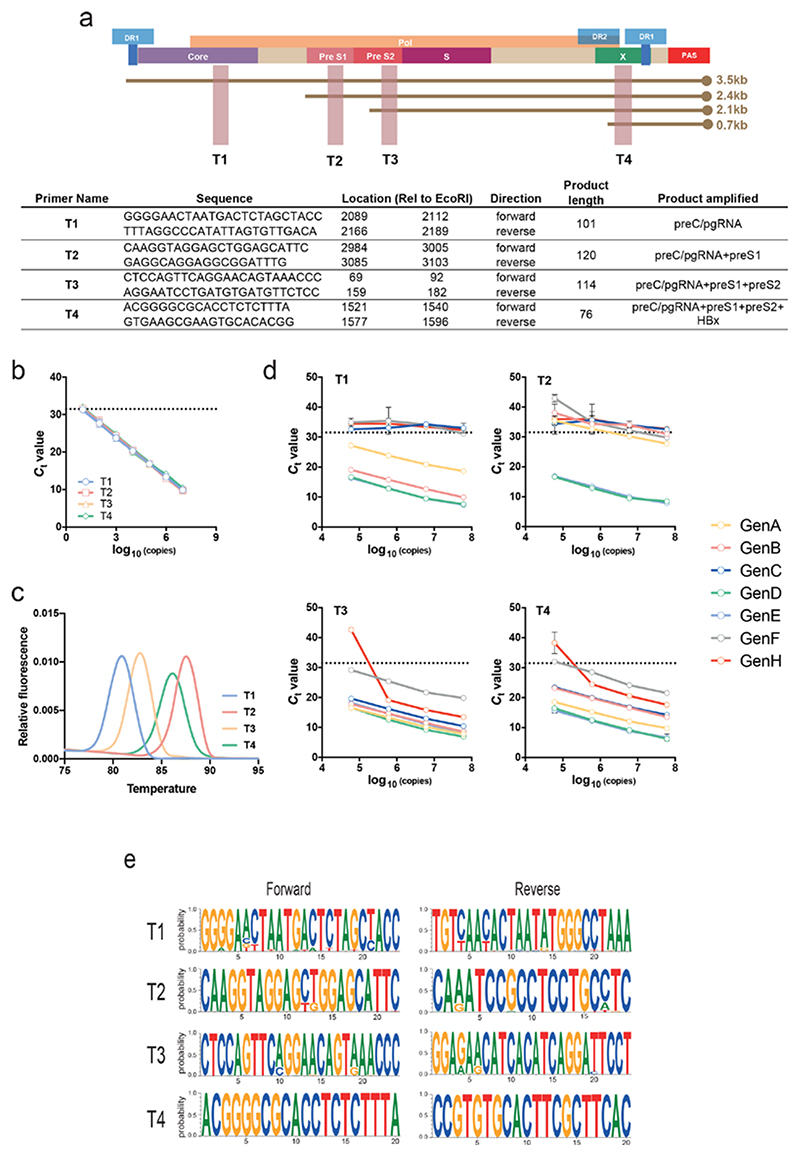
A qPCR-based approach to detect the major HBV RNAs. (a) Cartoon depicting HBV ayw genome organization showing the polydenylation site (PAS), the DR1 and DR2 motifs, the four major viral transcripts and the locations of the open reading frames for core, Pol, preS1, preS2, S and X. The primer sequences, their genome location, amplicon length and the transcripts amplified are shown. (b) A dilution series of a pHBV1.0 genotype D plasmid DNA of known copy number was used to evaluate the sensitivity and amplification efficiency of the T1–T4 primers. The dotted line depicts the limit of detection of the assay set to cycle 31 (≈50 copies). The four standard curves were superimposable with no significant differences in amplification efficiency. (c) Melting curve profiles of the PCR amplicons, showing four distinct Tm peaks. (d) Amplification of serial dilutions of plasmid HBV DNAs of known copy number bearing viral sequence from A to H genotypes with T1–T4 primers. (e) Sequence motif plots of the four primer sets aligned to HBV genotype D and E sequences in the HBV database (hbvdb.ibcp.fr).

To develop a qPCR-based method to quantify the major HBV RNAs we designed primers targeting short amplicons (76–120 bp) located in regions of the genome that correspond to overlapping viral RNAs. These amplicons target the pC/pg locus (T1); the pC/pg and preS1 loci (T2); the pC/pg, preS1 and preS2 loci (T3); and finally the pC/pg, preS1, preS2 and HBx loci (T4) (Fig. 1a). To assess the amplification efficiency of the primers we used a dilution series of a pHBV1.0 ayw genotype D plasmid and generated a standard curve where the copy number is plotted against *C*_t_ value for each primer pair. The primers showed comparable amplification efficiencies (90–100%) with a lower limit of detection of ~50 copies per reaction, equivalent to a detection threshold (*C*_t_ value) of ~31 cycles (Fig. 1b). To further validate the efficiency of the primers we spiked pHBV1.0 ayw into HepG2 cellular RNA and observed comparable amplification efficiency.

Analysing the melting curves for each amplicon showed a single peak with a distinct Tm (T1, 80 °C; T2, 88 °C; T3, 82 °C; and T4, 86 °C) (Fig. 1c), reflecting their differing lengths and GC content (Fig. 1a). These data enable the rapid identification of non-specific amplification products and act as an internal quality control. The specificity of the T1–T4 primers for their target was assessed by testing for cross-amplification of cloned T1–T4 amplicons. As expected, each primer pair only amplified its cognate target, with no amplification detected with any other plasmid (data not shown). We also evaluated the ability of the primers to amplify a panel of diverse HBV clones representing genotypes A–H (genotype G was not available) [18]. Primers T3 and T4 amplified products from all the diverse clones, although the amplification of genotype H was relatively insensitive (Fig. 1d). In contrast, the T1 and T2 primer pairs only amplified 4/7 (A, B, D and E) and 2/7 (D and E) genotypes, respectively (Fig. 1d), suggesting that the amplification of HBV genotypes other than D and E would require redesign of the T1 and T2 primers. Analysis of primer binding sites in the diverse HBV clones identified 0–2 mismatches in genotype E compared to the genotype D referent and binding sites showed lower conservation with the other genotypes studied (Table S1, available in the online version of this article). Genotype D and E clones showed similar PCR efficiencies for all primer pairs (Fig. 1d). Furthermore, alignment of the primers against a database of published HBV sequences (hbvdb.ibcp.fr) showed high conservation of the target regions among all known genotype D and E sequences (Fig. 1e).

It is unavoidable that there will be biases in the PCR amplification with different primers, despite the reaction efficiency being matched as closely as possible (90–100%), reflecting differences in cDNA synthesis, amplicon length, sequence and GC content and primer annealing efficiencies. However, by establishing consistent reaction conditions and analysis methods these biases will be uniform across the samples studied, and changes in the pattern of viral RNAs can be reliably ascribed to the samples under study.

### PCR quantification of HBV RNAs in HepG2.2.15 cells

We applied our PCR method to quantify viral RNAs in the well-characterized HepG2.2.15 cell line that carries two integrated copies of HBV DNA along with cccDNA [17, 23]. To ensure that our assay measures HBV RNAs and there is negligible carry-over of viral DNA, all samples were treated with DNase and screened in the PCR with or without the cDNA synthesis step. Samples did not amplify with any primers in the absence of cDNA synthesis. Comparison of different cDNA synthesis kits that used either a mixture of oligo dT/random hexamers or random hexamers alone gave comparable results. DMSO, a dipolar aprotic solvent, has been used extensively to induce and maintain the differentiation of numerous primary or tumour cell lines [24–26] and is routinely used for *in vitro* studies of HBV replication [17]. We assessed whether DMSO treatment alters the transcription pattern of HBV RNAs by isolating total cellular RNA from DMSO treated and untreated HepG2.2.15 cells for NB and qPCR analysis.

**Fig. 2:**
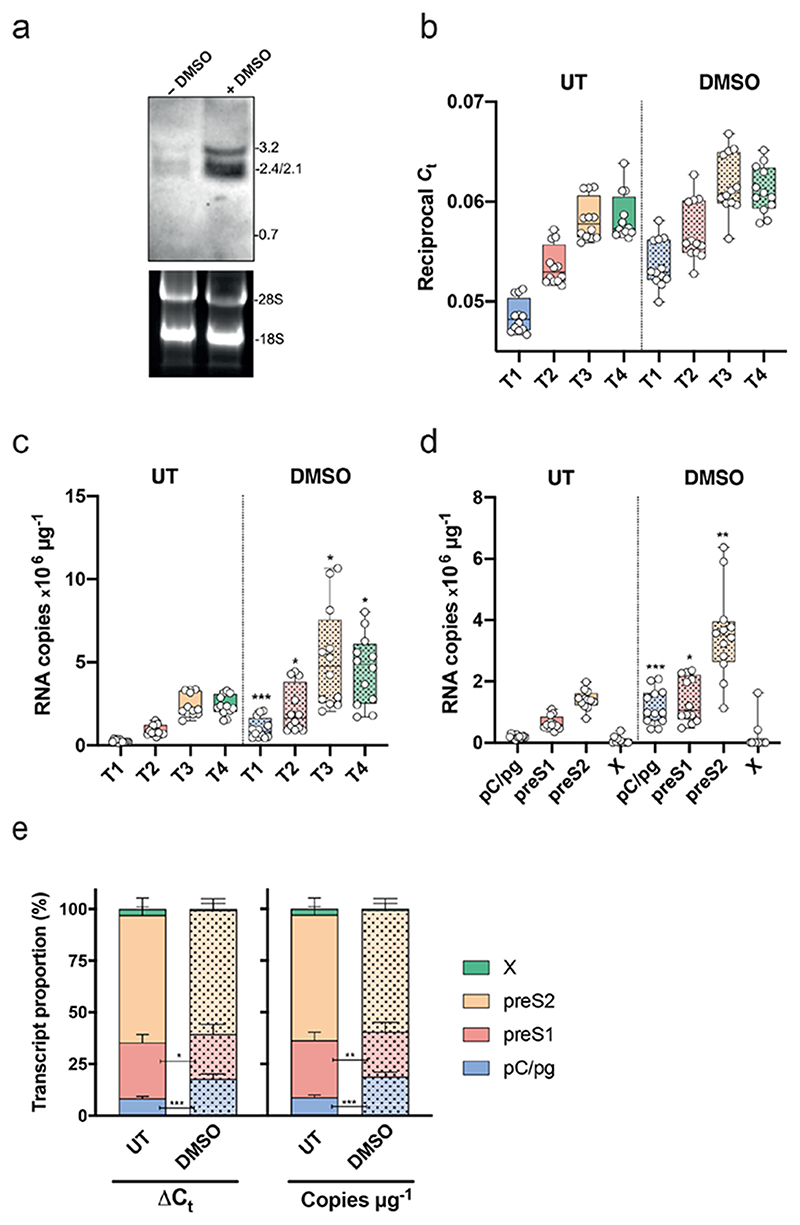
Validation of PCR assay with a HBV producer cell line. (a) Northern blot of total RNA prepared from DMSO treated or untreated HepG2.2.15 cells, where molecular weight of viral RNA and ribosomal RNA are shown. (b) Reciprocal *C*_t_ values obtained by T1, T2, T3 and T4 amplification of cDNA derived from DMSO-treated or untreated HepG2.2.15 cells. Data are presented as a box-and-whiskers plot with symbols denoting biological replicates. (c) RNA copy numbers inferred from the T1, T2, T3 and T4 *C*_t_ values. (Mann–Whitney test with Bonferroni correction; T1 *P*<0.0004, T2 *P*=0.0092, T3 *P*=0.018, T4 *P*=0.038). (d) Assigned copy numbers of each RNA transcript estimated by subtraction of the individual copy numbers in (c), comparison of RNAs from untreated and DMSO-treated HepG2.2.15 cells (Mann–Whitney test, pC/pgRNA *P*<0.0004, preS1 *P*=0.0144, preS2 *P*=0.002). (e) Proportion of viral RNAs as a percentage of the total in DMSO-treated and untreated HepG2.2.15 cells. Data are plotted as a stack bar and the error bars denote the standard deviation from the mean of replicate samples. The pattern of viral RNAs in DMSO-treated or untreated HepG2.2.15 cells was compared (pgRNA as ΔΔ*C*_t_ or copy number method: Mann–Whitney, *P*<0.0001 and *P*<0.0001, respectively; preS1 as ΔΔ*C*_t_ or copy number method: Mann–Whitney *P*=0.0145 and *P*=0.0083, respectively).

The NB showed negligible viral RNA in the untreated cells and DMSO increased both pC/pg and preS1/S2 RNAs (Fig. 2a). Due to the weak HBV RNA signals in the untreated sample, densitometric quantification of the NB was not reliable. A random hexamer directed cDNA synthesis was performed to generate an unbiased pool of template for subsequent PCR amplification. We observed an increasing amplification signal, with T3 >T2>T1, consistent with the primers amplifying multiple RNA species (Fig. 2b). We noted a similar amplification using T3 or T4 primers, suggesting negligible X RNA levels, consistent with the NB results. Cumulative viral RNA copy numbers were estimated using a standard curve for each primer pair and DMSO significantly increased all amplification products (T1–T4) (Mann–Whitney test) (Fig. 2c). The PCR readily detected viral RNAs in samples from untreated HepG2.2.15 cells, showing *C*_t_ values that were 2000-fold above the cut-off of the assay, demonstrating a more sensitive approach than NB analysis.

Since the primers co-amplify multiple transcripts, subtraction of the inferred copy number for selected primers can ascribe the relative copy number for each contributing RNA. Individual transcript analysis revealed that DMSO significantly increases the abundance of pC/pg, preS1 and preS2 RNAs, with no detectable HBx, confirming the results observed in the NB (Fig. 2d). Determining the relative expression of the major viral RNAs using raw *C*_t_ values (ΔΔ*C*_t_) or inferred viral RNA copy numbers showed preS2 to be the predominant RNA species in HepG2.2.15 cells (~60% of the inferred transcripts) (Fig. 2e). DMSO selectively increased the relative proportion of pC/pgRNAs, whether ΔΔ*C*_t_- (8.3 and 17.9% respectively) or copy number (8.8 and 18.8% respectively)-based methods of calculation were used (Fig. 2e), suggesting that DMSO preferentially increases the basal core promoter activity in HepG2 cells. Consequently, DMSO treatment reduced the preS1 proportion in both ΔΔ*C*_t_ or copy number analysis (from 27.5 to 21.7% and from 27.1 to 21.5%). Our observations highlight the sensitivity of this qPCR method to quantify HBV major RNAs. Published estimates of the efficiency of cDNA synthesis vary from 5 to 60% [27], but, assuming a conservative estimate of 5%, we can infer a threshold detection limit of 1000 RNA copies per reaction, a considerably more sensitive approach to quantify viral RNAs than NB.

### PCR amplification of viral RNAs from *de novo* HBV infections

**Fig. 3:**
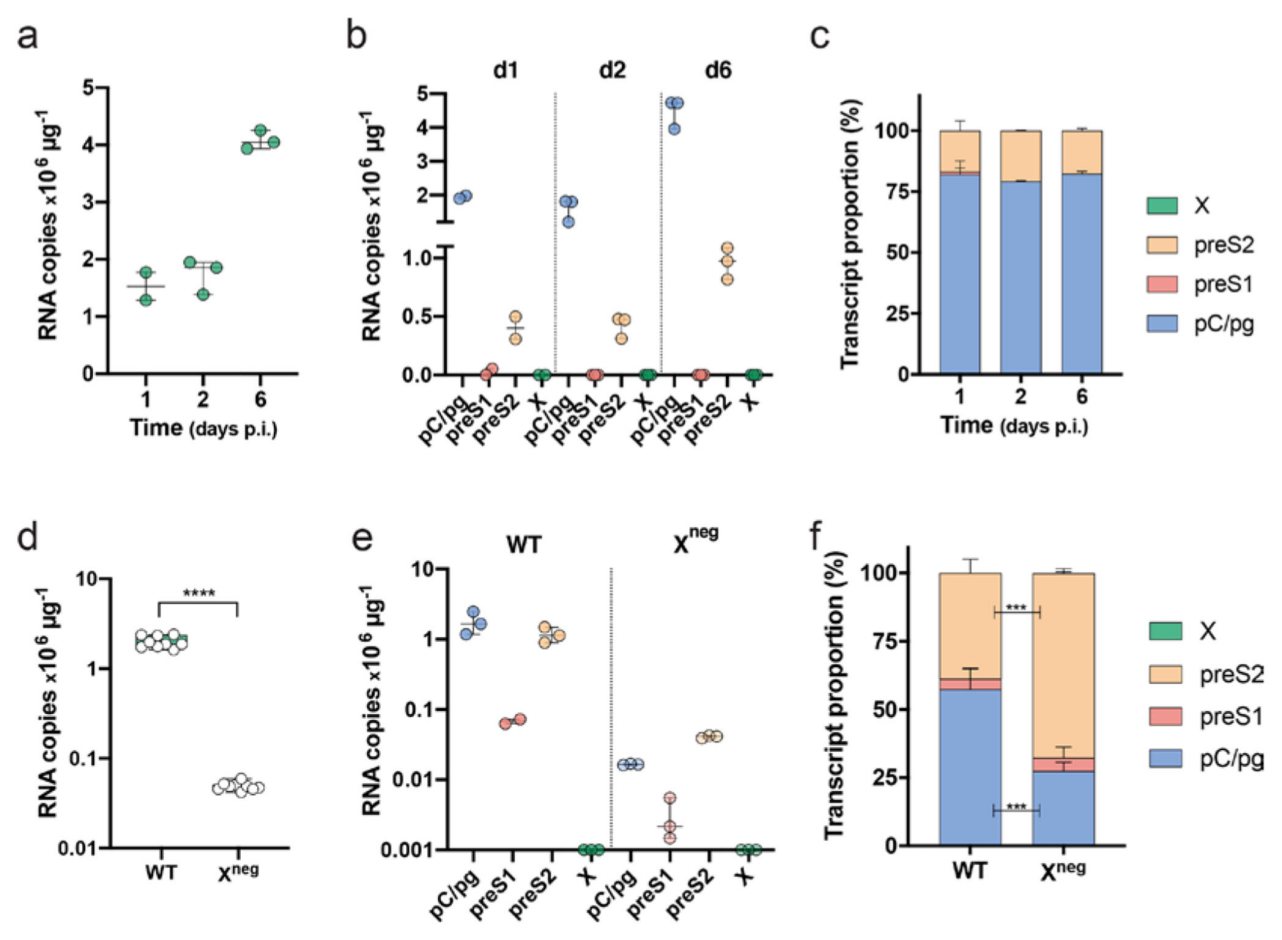
PCR enumeration of viral RNAs in HBV *de novo* infection. (a) Total viral RNAs and (b) relative RNA transcript copy numbers were inferred from T1, T2, T3 and T4 *C*_t_ values in samples from HBV-infected HepG2-NTCP cells after 1, 2 and 6 days. (c) Proportion of viral RNAs as a percentage of the total in HBV-infected HepG2-NTCP cells at 1, 2 and 6 days post-infection. Data are plotted as a stack bar and the error bars denote the standard deviation from the mean of replicate samples. (d) Total viral RNAs and (e) relative RNA transcript copy numbers inferred from T1, T2, T3 and T4 *C*_t_ values in samples from HBV WT and infected HepG2-NTCP cells after 7 days. (f) Proportion of viral RNAs as a percentage of the total in WT- and X^neg^-infected HepG2-NTCP cells, where the data are plotted as a stack bar and the error bars denote the standard deviation from the mean of replicate samples. (Mann–Whitney test; pC/pgRNA *P*<0.0004, preS2 *P*<0.0004.

HepG2-NTCP cells are one of the most permissive cell lines for HBV replication [17] and we used our qPCR method to assess the pattern of viral RNAs in newly infected cells. RNA was extracted from HBV-infected HepG2-NTCP cells after 1, 2 and 6 days, and viral RNAs quantified by PCR. Total viral RNAs, as measured with the T4 primers, increased over the course of infection (Fig. 3a-b), with the pC/pg and preS2 transcripts comprising ~80 %and~20% of the total viral RNAs, respectively (Fig. 3c). Furthermore, the pattern of viral RNAs showed no significant change over the time course of this experiment (Fig. 3c). This pattern of viral transcripts is representative of HBV-infected DMSO-arrested HepG2-NTCP cells. We noted variable infection efficiencies between independent experiments where the total viral RNA burden can vary by up to 1-log, however, pre-core/pre-genomic is the dominant RNA species. We failed to detect any X RNA in the infected HepG2-NTCP cells, consistent with HepG2.2.15 data and earlier published work [17]. To confirm that the T4 primers can amplify X RNA we used a HepaRG cell line that expresses HBx under a Tet-inducible promoter [21]. Inducing HBx expression boosted T4 amplification by >6 *C*_t_, reflecting a >64-fold induction in X RNA, demonstrating the specificity of the T4 primers. It is interesting to observe the different pattern of viral RNAs in HepG2.2.15 cells and *de novo*- infected HepG2-NTCP cells, which may reflect differential chromatinization and promoter usage in integrated viral DNA versus cccDNA episomes.

The HBx protein regulates viral transcription and viruses engineered with a stop codon in their HBx open reading frame (X^neg^) have been reported to be transcriptionally inactive as assessed using NB [21]. We were interested to confirm these observations using our qPCR assay and analysed RNA samples from wild-type (WT)- and HBV (X^neg^)-infected HepG2-NTCP cells at 7 days post-infection. We observed a significant 100-fold reduction in pC/pgRNA levels in the X^neg^-infected cells compared to WT infection (Fig. 3d) and this was accompanied by a>200-fold reduction in HBeAg expression (WT 129±13 and X^neg^ 0.6±0.1 PEI U ml^−1^). There was an approximate 22-fold reduction in preS2 RNA levels in X^neg^-infected cells compared to WT (2×10^6^ and 9×10^4^ copies μg^−1^, respectively) (Fig. 3e).The ability of X^neg^ virus to transcribe low levels of preS2 RNA is consistent with the detection of HBsAg (WT 8.8±2.4 and X^neg^ 0.6±0.3 IU ml^−1^), suggesting that this X^neg^ virus is not transcriptionally silent and can transcribe low levels of viral RNAs, with preS2 comprising the major species (Fig. 3f).

Regulation of HBV transcription is not well understood and most reports studying transcription only quantify total HBV RNA or pgRNA. Here we describe a simple qPCR-based approach that accurately quantifies the relative abundance of the major HBV RNA transcripts. Although some work has been reported in this area [14, 28], these studies have been compromised by the differing lengths of the viral amplicons and undefined efficiencies of the PCR reactions. Our qPCR-based observations agree with a study where a combined 5” RACE/RNA-Seq approach was used to identify the relative abundance and transcription start sites of HBV RNA [29]. Two recent studies used PCR approaches to compare the relative levels of pC/pg RNA to total viral RNA in biopsy samples from chronic HBV-infected subjects, concluding that pc/pgRNA levels vary and the ratio of pC/pg : total RNA declines in chronic disease [14, 30]. Together, these studies suggest that there are differences in the pattern of HBV transcription and that a simple and cost-effective assay to measure the relative levels of the major viral RNAs would be a useful tool to understand the host pathways that regulate HBV epigenome and assess antiviral agents [31]. Our study demonstrates that a relatively simple differential PCR approach can provide useful insights into the transcriptional activity of HBV.

## Supplementary Material

Supplementary Table

## Five reasons to publish your next article with a Microbiology Society journal

1. The Microbiology Society is a not-for-profit organization.
2. We offer fast and rigorous peer review – average time to first decision is 4–6 weeks.
3. Our journals have a global readership with subscriptions held in research institutions around the world.
4. 80% of our authors rate our submission process as ‘excellent’ or ‘very good’.
5. >Your article will be published on an interactive journal platform with advanced metrics.

**Find out more and submit your article at microbiologyresearch.org**.

## Abbreviations

cccDNA: covalently closed circular DNA
DMEM: Dulbecco’s Modified Eagles Medium
DMSO: Dimethyl sulphoxide
FBS: fetal bovine serum
HBeAg: Hepatitis B Virus e antigen
HBsAg: Hepatitis B Virus s antigen
HBV: Hepatitis B Virus
HBx: Hepatitis B Virus x protein
MOI: Multiplicity of infection
NB: Northern blot
PBS: Phosphate-buffered saline
pC: pre-core
pg: pre-genomic
WT: Wild type
