# Supplementary material for: A PCR assay to quantify patterns of HBV transcription
Source: J Gen Virol. 2019 Dec 17;102(3):001373. doi: 10.1099/jgv.0.001373 (PMC7610515; doi:10.1099/jgv.0.001373)
Supplement: Supplementary material 1 [file jgv-102-1373-s001.pdf]

	T1		T2		T3		T4	
	F	R	F	R	F	R	F	R
Genotype A	GGGGAA**T**T**G**ATGACTCTAGCTACC	TTTA**AA**CCCAT**G**TTAGT**A**TT**A**ACA	C**C**AGGTAGGAG**TG**GGAGCATTC	GAGGCAGGAGG**A**GGA**A**TTG	CTCCAGTTCAGGAACAGTAAACCC	AGGAATCCTGATGTGATGTTCTCC	ACGGGGCGCACCTCTCTTTA	GTGAAGCGAAGTGCACACGG
Genotype B	GG**T**GA**GT**TAATGA**A**TCTAGC**C**ACC	TT**C**AGGCCCATATTA**AC**GTTGACA	CAAGGT**G**GGAG**TG**GGAGCATTC	GAGGCAGGAGG**A**GGA**GC**TG	CTCCAGTTCAGGAACAGT**G**A**G**CCC	AGGA**G**TCCTGATG**C**GATGTTCTCC	ACGGGGCGCACCTCTCTTTA	GTGAAGCGAAGTGCACACGG
Genotype C	GG**T**GA**GT**T**G**ATGA**A**TCT**G**GC**C**ACC	TTTAGGCCCATATTA**ACA**TTGACA	**TC**AGGTAGGAGC**G**GGAGCATTC	GAGGCAGGAGG**A**GG**TGC**T**A**	CTCCAGTTC**C**GGAACAGTAAACCC	AGGAATCCTGATGT**TG**TG**C**TCTCC	ACGGGGCGCACCTCTCTTTA	GTGAAGCGAAGTGCACACGG
Genotype D	GGGGAACTAATGACTCTAGCTACC	TTTAGGCCCATATTAGTGTTGACA	CAAGGTAGGAGCTGGAGCATTC	GAGGCAGGAGGCGGATTTG	CTCCAGTTCAGGAACAGTAAACCC	AGGAATCCTGATGTGATGTTCTCC	ACGGGGCGCACCTCTCTTTA	GTGAAGCGAAGTGCACACGG
Genotype E	GGGGAACTAATGACTCTAGCTACC	TTTAGGCCCATATTAGT**A**TTGACA	CAAGGTAGGAG**TG**GGAGCATTC	GAGGCAGGAGGCGGAT**C**TG	CTCCAGTTC**C**GGAACAGT**G**AACCC	AGGAATCCTGATGTGATG**C**T**T**TCC	ACGGGGCGCACCTCTCTTTA	GTGAAGCGAAGTGCACACGG
Genotype F	GG**T**GA**GT**TAATGACT**T**T**G**GCT**T**CC	TTTAGGCCCA**TG**TTAGT**A**TTGACA	CAAGGTAGGAG**TG**GGAG**G**AT**A**C	GA**A**GCAGGAGGCGGAT**C**TG	CTCCAGTTCAGAGACACAGAACCC	AGGA**G**TCCTGATGTGATGTT**G**TCC	ACGGG**T**CGCACCTCTCTTTA	GTGAAGCGAAGTGCACACGG
Genotype H	GG**T**GA**GT**T**G**ATGAC**CT**T**G**GCT**T**CC	TTTAGGCCCAT**G**TTAGT**A**TTGACA	CAAGGTAGGAG**TG**GGAG**GC**TTC	GA**A**GCAGGAGG**T**GGAT**C**TG	CTCCAGTTCAGAAACACAGAACCC	AGGA**G**TCCTGATGTGATGTTCTCC	ACGGG**T**CGCACCTCTCTTTA	GTGAAGCGAAGTGCACACGG
